# Privacy-Aware Continual Self-Supervised Learning on Multi-Window Chest Computed Tomography for Domain-Shift Robustness

**DOI:** 10.3390/bioengineering13010032

**Published:** 2025-12-27

**Authors:** Ren Tasai, Guang Li, Ren Togo, Takahiro Ogawa, Kenji Hirata, Minghui Tang, Takaaki Yoshimura, Hiroyuki Sugimori, Noriko Nishioka, Yukie Shimizu, Kohsuke Kudo, Miki Haseyama

**Affiliations:** 1 Graduate School of Information Science and Technology, Hokkaido University, N-14, W-9, Kita-ku, Sapporo 060-0814, Hokkaido, Japan; tasai@lmd.ist.hokudai.ac.jp; 2 Education and Research Center for Mathematical and Data Science, Hokkaido University, N-12, W-7, Kita-ku, Sapporo 060-0812, Hokkaido, Japan; guang@lmd.ist.hokudai.ac.jp; 3 Faculty of Information Science and Technology, Hokkaido University, N-14, W-9, Kita-ku, Sapporo 060-0814, Hokkaido, Japan; togo@lmd.ist.hokudai.ac.jp (R.T.); ogawa@lmd.ist.hokudai.ac.jp (T.O.); 4 Faculty of Medicine, Hokkaido University, N-15 W-7, Kita-ku, Sapporo 060-8648, Hokkaido, Japan; khirata@pop.med.hokudai.ac.jp (K.H.); toumeiki@hs.hokudai.ac.jp (M.T.); nnishioka@pop.med.hokudai.ac.jp (N.N.); yshimizu7298@med.hokudai.ac.jp (Y.S.); kkudo@med.hokudai.ac.jp (K.K.); 5 Faculty of Health Sciences, Hokkaido University, N-12, W-5, Kita-ku, Sapporo 060-0812, Hokkaido, Japan; takaaki.ysm@med.hokudai.ac.jp (T.Y.); sugimori@hs.hokudai.ac.jp (H.S.)

**Keywords:** self-supervised learning, continual self-supervised learning, feature distillation, latent replay, chest CT image

## Abstract

We propose a novel continual self-supervised learning (CSSL) framework for simultaneously learning diverse features from multi-window-obtained chest computed tomography (CT) images and ensuring data privacy. Achieving a robust and highly generalizable model in medical image diagnosis is challenging, mainly because of issues, such as the scarcity of large-scale, accurately annotated datasets and domain shifts inherent to dynamic healthcare environments. Specifically, in chest CT, these domain shifts often arise from differences in window settings, which are optimized for distinct clinical purposes. Previous CSSL frameworks often mitigated domain shift by reusing past data, a typically impractical approach owing to privacy constraints. Our approach addresses these challenges by effectively capturing the relationship between previously learned knowledge and new information across different training stages through continual pretraining on unlabeled images. Specifically, by incorporating a latent replay-based mechanism into CSSL, our method mitigates catastrophic forgetting due to domain shifts during continual pretraining while ensuring data privacy. Additionally, we introduce a feature distillation technique that integrates Wasserstein distance-based knowledge distillation and batch-knowledge ensemble, enhancing the ability of the model to learn meaningful, domain-shift-robust representations. Finally, we validate our approach using chest CT images obtained across two different window settings, demonstrating superior performance compared with other approaches.

## 1. Introduction

Medical image analysis is crucial to clinical decision-making for diagnostic support [1,2]. Its performance has been dramatically improved by the emergence of deep learning-based supervised learning (SL) [3,4,5]. While automating parts of the diagnostic process can enhance the quality and efficiency of clinical judgment, models deployed in critical medical settings must be designed to simultaneously achieve high accuracy and function robustly, with generalizability across diverse datasets and conditions. However, SL is mainly limited by the significant shortage of large-scale, accurately annotated medical image datasets [6,7,8], and this scarcity is exacerbated because annotating medical data, which must balance privacy protection with accuracy, requires extensive expertise and considerable effort. Consequently, the model efficiency relies heavily on the availability of high-quality annotated data.

As an approach for addressing this data-scarcity issue, self-supervised learning (SSL) has garnered attention [9,10,11,12]. In SSL, a model is first pretrained using unlabeled data and then fine-tuned with a small amount of labeled data. Additionally, reports reveal that SSL achieves outstanding performance while effectively reducing labeling costs [13,14,15]. However, SSL still exhibits a key limitation: it lacks generalizability in real-world healthcare environments because the dynamic nature of clinical settings causes changes in the data distributions of medical images over time, resulting in domain shifts [16,17,18]. This shift stems from differences across medical institutions, imaging equipment, and diagnostic objectives, resulting in high diversity across medical images. A prominent example is in chest computed tomography (CT), where images often comprise multiple domains, such as the mediastinal and lung window settings, each optimized for distinct clinical-observation purposes [19,20]. In these dynamic clinical settings, data with different characteristics arrive sequentially, necessitating the continuous handling of domain shifts. However, conventional SSL relies on a joint training scenario, where a model is trained only after collecting a large amount of unlabeled data. Consequently, the model cannot flexibly adapt to new data without expensive retraining [21,22]. Furthermore, satisfying this premise in real-world scenarios is often challenging owing to the high computational costs of retraining and strict privacy constraints [23,24].

In response to these challenges, continual SSL (CSSL) was recently applied to medical imaging [25]. This approach involves allocating data with varying characteristics across multiple training stages for continual pretraining. Particularly, maintaining data-distribution diversity during this pretraining process enables the acquisition of rich feature representations that are beneficial for subsequent fine-tuning. Notably, CSSL mitigates data interference that typically occurs when integrating different modalities or diverse domains within joint SSL frameworks [26,27]. Additionally, CSSL achieves good accuracy and generalizability compared to representative supervised continual learning (SCL) paradigms [28,29]. Therefore, CSSL is projected to address labeling-cost reduction and dynamic-environment domain shifts.

Notably, the primary challenge of CSSL is catastrophic forgetting [30,31], which occurs when a model overwrites or forgets previously acquired knowledge while learning new concepts. To mitigate this issue, CSSL conventionally adopts experience-replay-based approaches [32,33] from SCL. In this approach, a portion of the original images is stored in a memory buffer (*B*), enabling the model to retain and revisit past knowledge during later sequential training stages. This strategy is highly versatile, exhibiting applicability in a wide range of scenarios compared with other SCL approaches. However, in the medical-data context, the retention of past datasets is often complicated by privacy concerns [34,35]. In the SCL field, latent replay (LR)-based approaches have been introduced to address catastrophic forgetting. These approaches achieve privacy preservation by storing the activations of intermediate layers in neural networks (NNs) and replaying them when learning new knowledge [36,37]. Specifically, they store feature representations instead of preserving the original images, leveraging them in subsequent learning stages to ensure data privacy. Nevertheless, LR remains largely unexplored within the CSSL context, necessitating the exploration of a novel LR-based CSSL framework.

To satisfy the aforementioned research gap, we propose a novel CSSL framework that simultaneously addresses domain shifts in dynamic environments while effectively mitigating catastrophic forgetting under privacy-constraint conditions. The proposed framework maintains a *B* that stores only the feature representations of past data, enabling the continual pretraining of rich representations while preserving data privacy and distribution diversity. To realize this, we develop a feature distillation method that integrates Wasserstein distance (WD)-based knowledge distillation (WKD) with a batch-knowledge ensemble (BKE). While WKD enforces distributional alignment between replayed and mini-batch features, BKE aggregates feature representations to enhance consistency and reduce domain interference. This unified WKD-BKE design facilitates the learning of robust, generalizable features across multiple domains, exhibiting suitability for privacy-conscious CSSL. Further, to evaluate the effectiveness of the proposed framework, we pretrain a model using chest CT images acquired under two different window settings and evaluate its performance on two distinct public CT-image datasets. Extensive experiments demonstrate that the proposed framework consistently outperforms other approaches, achieving superior robustness and performance.

The contributions of our study are summarized below.

- We propose a novel LR-based CSSL framework to ensure data privacy and effectively address catastrophic forgetting during pretraining with chest CT images across two domains.
- We introduce a novel WKD-BKE-integrated feature distillation method to simultaneously enable robust feature-representation learning and mitigate data interference.
- Our extensive experiments reveal that our method outperforms state-of-the-art approaches on two public chest-CT-image datasets.

The remainder of this paper is organized as follows: Section 2 discusses the extant studies, Section 3 describes the details of the proposed CSSL framework, and Section 4, Section 5 and Section 6 present the experiments, discussion, and conclusions, respectively.

## 2. Related Studies

### 2.1. Self-Supervised Learning for Addressing Domain Shifts

SSL has recently garnered significant attention in medical image analysis, which is characterized by limited annotated data. SSL has been applied across various modalities, including CT [38,39,40], magnetic resonance imaging (MRI) [41,42], fundus imaging [43,44], and ultrasound localization microscopy (ULM) [45,46]. However, domain shifts due to differences in imaging equipment, acquisition protocols, and diagnostic objectives typically compromise model reliability and robustness. To address this, multi-domain pretraining under the joint-training condition has been explored.

In CT-based studies, Wolf et al. [38] proposed a masked modeling-based SSL method for convolutional NNs. The authors pretrained the model on a large-scale chest-CT dataset obtained from multiple medical institutions and demonstrated its effectiveness using classification tasks. Similarly, Jiang et al. [39] investigated the robustness of SSL to domain shifts for tumor segmentation in non-small-cell lung cancer CT. Employing MRI, Fiorentino et al. [42] introduced an intensity-based self-supervised domain-adaptation approach for intervertebral disc segmentation. This approach effectively reduced annotation costs and improved generalizability across scanners with heterogeneous acquisition settings. Mojab et al. [44] employed fundus imaging to demonstrate the superior adaptability of SSL-pretrained models, which were trained on multi-device datasets to unseen domains in glaucoma detection. Yu et al. [45] addressed domain shifts in ultrasound localization microscopy caused by differences between simulated and real ultrasound data, imaging depth, and noise characteristics, and proposed a semi-supervised approach to mitigate cross-domain discrepancies in microbubble signal representations.

Overall, these studies demonstrated that SSL-based pretraining represents an effective strategy for mitigating domain shifts in medical image analysis. However, studies revealed that representation learning across different modalities and domains can interfere with each other during pretraining, primarily because of their substantial differences, which ultimately results in data interference [26,27]. Furthermore, in the application of SSL to clinical settings that are characterized by dynamically changing data distributions, models often require retraining on the entire dataset. Such retraining requires considerable computational resources. Moreover, the often limited access to all data due to privacy constraints poses significant challenges for clinical applications.

### 2.2. Continual Self-Supervised Learning for Addressing Domain Shifts

In recent years, the application of CSSL has primarily focused on natural images, exploring its ability to handle incrementally arriving data. This capability is especially relevant in real-world settings where it is often impractical to assemble all data in advance. Fini et al. [23] experimented on DomainNet [47] involving sequential learning across multiple domains, demonstrating that CSSL outperformed major SSL and effectively addressed domain shifts. Furthermore, Hu et al. [29] experimentally demonstrated on DomainNet that combining CSSL with simple SCL strategies, such as experience replay or parameter regularization [48], can significantly mitigate performance degradation, even under substantial distributional shifts. This and related studies provide valuable insights, collectively indicating that CSSL operates effectively in real-world scenarios, even in the presence of domain shifts.

These advances motivated a growing interest in the application of CSSL to medical imaging analysis. For instance, Ye et al. [26] and Yao et al. [49] proposed methods for enhancing robustness and scalability in cross-modality learning. These approaches leverage experience replay and feature distillation [50,51] to efficiently integrate new modalities while suppressing the representational interference that often arises in conventional SSL. However, these studies were limited to demonstrating effectiveness across multiple modalities and did not sufficiently examine applicability to multiple domains (i.e., domain shift within a single modality). Conversely, a CSSL method was recently proposed [27] using chest CT images acquired under heterogeneous scanning conditions. The method involves the introduction of an experience-replay-based approach for balancing sample diversity and representativeness within *B*. This design enabled the acquisition of domain-invariant feature representations during continual pretraining. The authors deployed a COVID-19 classification task to demonstrate the effectiveness of CSSL in mitigating representational interference in conventional SSL while maintaining robustness across diverse domains.

These medical-imaging studies generally adopted experience-replay-based approaches, which store past image samples in *B* and reuse them in subsequent pretraining stages, thereby mitigating catastrophic forgetting. Experience replay has demonstrated improved robustness in continual pretraining across multiple modalities and domains in medical imaging. Although this strategy is highly versatile and applicable to a wide range of scenarios, retaining past datasets is often impractical owing to concerns about preserving medical data privacy. In contrast to experience replay, recent privacy-aware continual learning frameworks have investigated LR mechanisms, which store intermediate feature representations instead of original images. These approaches aim to reduce privacy risks by avoiding the direct storage of raw data. In many existing methods, additional operations such as nonlinear transformations, compression, or quantization are applied to the stored representations in order to suppress input reconstruction via inversion models. In this study, we propose a novel CSSL framework for chest CT images spanning two domains, integrating an LR-based approach. To the best of our knowledge, the integration of LR into a CSSL framework has not been extensively explored in prior work. Accordingly, as an initial investigation, we adopt a design choice in which no additional transformations are applied to the stored feature representations. Dissimilar to conventional experience replay, our method only retains feature representations in *B* rather than the raw data, thereby achieving privacy-preserving continual pretraining while enabling the progressive acquisition of more expressive representations.

## 3. Privacy-Aware Continual Self-Supervised Learning Integrating Latent Replay and Feature Distillation

**Figure 1:**
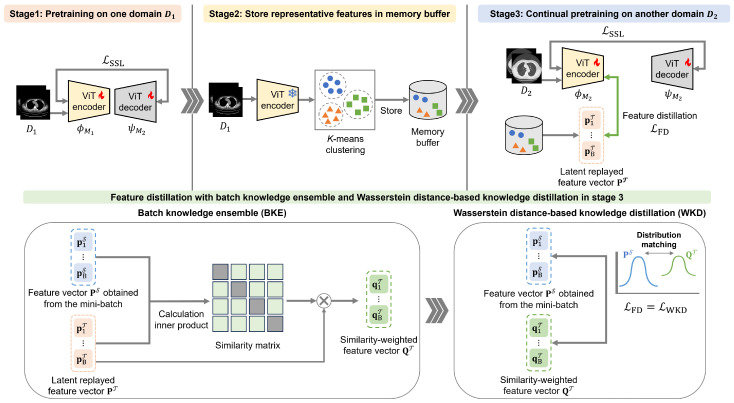
Overview of the proposed continual self-supervised learning (CSSL) framework.

Our CSSL framework comprises a three-stage sequential process for pretraining the vision transformer (ViT) [52] encoder employed in subsequent downstream tasks. In the first stage, SSL is performed using the initial dataset, $D_{1}$, from one chest-CT-image domain. In the second stage, selected feature representations from $D_{1}$ are stored in *B* to preserve data diversity and privacy. In the third stage, SSL is performed again using the next (second-domain) dataset, $D_{2}$, from another domain. In this third stage, feature distillation involving WKD-BKE integration is performed using replayed features from *B*. Afterward, fine-tuning is performed using labeled data. Figure 1 shows an overview of the proposed CSSL framework.

### 3.1. Stage 1: Self-Supervised Learning on the First-Domain Dataset

The first pretraining stage proceeds with a model, $M_{1}$, using $D_{1}$. The masked autoencoder (MAE) method [53] is employed to learn feature representations from the input data. This pretraining task uses a reconstruction objective that compares the original patches with their reconstructed masked counterparts, enabling the model to learn meaningful representations from unlabeled data. The network architecture consists of an encoder, $\phi_{M_{1}}$, and its corresponding decoder, $\psi_{M_{1}}$.

In this process, each image with *C* channels is divided into *n* patches of size (V,V), which are collectively represented as $X_{V}\in R^{n\times (V^{2}\times C)}$. A masking rate *r* is then applied to $X_{V}$, and m=n×r patches are randomly selected as the patches to be masked, which are denoted as $X_{m}$. Next, all patches are converted into a sequence of tokens using a tokenizer, $T_{M_{1}}$. Thereafter, the tokens corresponding to the n−m unmasked patches are fed into the encoder $\phi_{M_{1}}$ to generate latent feature representations. The decoder, $\psi_{M_{1}}$, reconstructs the original patch contents of $X_{m}$ into reconstructed patches $Y_{m}$ by predicting their pixel values using the feature representations obtained from the encoder, together with the embeddings of $X_{m}$ provided by $T_{M_{1}}$. Afterward, the model is optimized to minimize the mean squared error between $X_{m}$ and $Y_{m}$ as follows:

(1) $$L_{\mathrm{SSL}}=\frac{1}{m\times V^{2}\times C}\left|\right|Y_{m}-X_{m}{\left|\right|}_{2}^{2}.$$

This first stage terminates with the training of $M_{1}$ to capture comprehensive feature representations from $D_{1}$. This trained ($M_{1}$) model is subsequently employed in the third CSSL stage, which integrates $D_{1}$ and $D_{2}$.

### 3.2. Stage 2: Sampling Features in the Memory Buffer

The second stage involves the selection of features stored in *B*. This process is crucial to capturing data-distribution changes across different stages and mitigating catastrophic forgetting. Additionally, the utilization of these selected features in the third stage ensures data privacy.

In this work, the output of the final encoder layer of a pretrained ViT encoder $\phi_{M_{1}}$ is used as the feature representation of $D_{1}$ in the first stage. These features integrate global contextual information across all tokens through self-attention, which can facilitate clustering and sample selection [54]. First, these feature representations are divided into N×α clusters. Finally, N×β features closest to the cluster centers are selected and stored in *B*. In this algorithm, *N* denotes the number of images in $D_{1}$, and α and β are control parameters for the sampling ratio.

### 3.3. Stage 3: Continual Self-Supervised Learning with Feature Distillation Using the Second-Domain Dataset

Following the SSL pretraining of model $M_{1}$ in the first stage, another model, $M_{2}$, is pretrained in the third stage using the MAE method. Notably, $M_{2}$ is trained using $D_{2}$ and the replayed features from *B* in the second stage. Furthermore, WKD-BKE-integrated feature distillation enables $M_{2}$ to retain the knowledge acquired in the first stage while learning new representations for the second domain.

#### 3.3.1. Wasserstein Distance-Based Knowledge Distillation

WKD facilitates knowledge retention by aligning the feature distributions of $M_{2}$ and $M_{1}$. Specifically, it compares the $M_{1}$-associated feature representations (replayed from *B*) with those generated by $M_{2}$ in the third stage.

We consider a feature map derived from the feature representations, as follows: let the spatial height, width, and channel number of this feature map be *h*, *w*, and *l*, respectively. Next, the feature map is transformed into a matrix, $F\in R^{l\times d}$, where d=h×w, and the *i*-th column $f_{i}\in R^{l}$ represents the spatial features. Thereafter, we estimate the first and second moments, $\mu =\frac{1}{d}\sum_{i}f_{i}$ and $\Sigma =\frac{1}{d}\sum_{i}\left(f_{i}-\mu \right){\left(f_{i}-\mu \right)}^{T}$, respectively, from these features. The feature distribution of the input images is modeled as a Gaussian distribution parameterized by the mean vector, μ, and covariance matrix, Σ, as follows:

(2) $$N\left(\mu ,\Sigma \right)=\frac{1}{{\left|2\pi \Sigma \right|}^{1/2}}\exp\left\{-\frac{1}{2}{\left(f-\mu \right)}^{T}\Sigma^{-1}\left(f-\mu \right)\right\},$$

where |·| is the matrix determinant. Additionally, we define the teacher’s and student’s feature distributions as $N^{T}≜N\left(\mu^{T},\Sigma^{T}\right)$ and $N^{S}$, respectively. The continuous WD between the two Gaussian distributions is expressed by the following:

(3) $$D_{\mathrm{WD}}\left(N^{T},N^{S}\right)=\underset{q}{\inf}\int_{R^{l}}\int_{R^{l}}\left|\right|f^{T}-f^{S}{\left|\right|}^{2}q\left(f^{T},f^{S}\right)df^{T}df^{S},$$

where inf represents the infimum, which is the greatest lower bound, $f^{T}$ and $f^{S}$ are Gaussian variables, and $\left|\right|\cdot {\left|\right|}^{2}$ denotes the Euclidean distance. The joint distribution, *q*, is constrained such that its marginal distributions correspond to $N^{T}$ and $N^{S}$. Thus, to minimize this equation and following [55,56], we define the WKD loss function, $L_{\mathrm{WKD}}$, as follows:

(4) $$L_{\mathrm{WKD}}=\gamma D_{\mathrm{mean}}\left(\mu^{T},\mu^{S}\right)+D_{\mathrm{cov}}\left(\Sigma^{T},\Sigma^{S}\right).$$

Here, $D_{\mathrm{mean}}\left(\mu^{T},\mu^{S}\right)=\left|\right|\mu^{T}-\mu^{S}{\left|\right|}^{2}$ and $D_{\mathrm{cov}}\left(\Sigma^{T},\Sigma^{S}\right)=\left|\right|\delta^{T}-\delta^{S}{\left|\right|}^{2}$, where $\delta^{T}$ and $\delta^{S}$ are the standard deviation vectors formed from the square root of the diagonal elements of $\Sigma^{T}$ and $\Sigma^{S}$, respectively. Diagonal covariance matrices were employed for their robustness in estimating high-dimensional features as well as their computational efficiency [55,57]. To balance the roles of the mean and covariance, we introduce a mean–covariance ratio hyperparameter, γ. By computing $L_{\mathrm{WKD}}$, we enable the feature distribution of $M_{2}$ to align with that of $M_{1}$, thereby mitigating data interference due to inter-stage domain shifts (data-distribution differences across stages).

#### 3.3.2. Batch Knowledge Ensemble

We apply the BKE approach to enable $M_{2}$ to concurrently achieve robust learning while knowledge retention from the first stage. This approach enables feature distillation based on the similarity between *B* feature representations, $P^{T}$, randomly replayed from the memory buffer and the feature representations, $P^{S}$, within the mini-batch generated by the encoder, $\phi_{M_{2}}$ of $M_{2}$, in the third stage. Therefore, knowledge is propagated and ensembled via the affinity between the feature representations replayed from *B* and those generated by $M_{2}$ within mini-batches in the third stage.

Let the batch size, number of tokens, and embedding dimension be *B*, *T*, and *E*, respectively. Thus, $P^{T},P^{S}\in R^{B\times T\times E}$. First, we obtain the similarity matrix, $A\in R^{B\times T\times T}$, by calculating the similarities between the replayed feature representations, $\left\{p_{1}^{T},\ldots ,p_{B}^{T}\right\}$, retrieved from *B*, and the encoded visual features, $\left\{p_{1}^{S},\ldots ,p_{B}^{S}\right\}$, extracted from a mini-batch of *B* images, as follows:

(5) $$A_{i,j}=\left({\hat{p}}_{i}^{T⊤}{\hat{p}}_{j}^{S}\right).$$

In this equation, each feature representation is denoted as $p_{i}^{T},p_{j}^{S}\in R^{T\times E}$, where ${\hat{p}}_{i}^{T}=p_{i}^{T}/{∥p_{i}^{T}∥}_{2}$ and ${\hat{p}}_{j}^{S}=p_{j}^{S}/{∥p_{j}^{S}∥}_{2}$ represent the normalized feature representations. The indices, *i* and *j*, refer to the tokens within each mini-batch sample and replayed memory, respectively. Next, we normalize $A\in R^{B\times T\times T}$ as follows:

(6) $${\hat{A}}_{i,j}=\frac{\exp\left(A_{i,j}\right)}{\sum_{j\neq i}\exp\left(A_{i,j}\right)},\forall i\in \left\{1,\ldots ,B\right\}.$$

To prevent the excessive propagation and aggregation of noisy predictions, the optimized feature representation, Q, is generated as a weighted sum of feature representation $P^{T}$ and propagated probability matrix $\hat{A}P^{T}$ as follows:

(7) $$Q=\omega \hat{A}P^{T}+\left(1-\omega \right)P^{T}.$$

Notably, propagation can proceed multiple times to generate Q for feature distillation:

(8) $$\begin{array}{cc} Q_{\left(t\right)} & =\omega \hat{A}Q_{\left(t-1\right)}+\left(1-\omega \right)P^{T},\\ & ={\left(\omega \hat{A}\right)}^{t}P^{T}+\left(1-\omega \right)\sum_{i=0}^{t-1}{\left(\omega \hat{A}\right)}^{i}P^{T}, \end{array}$$

where ω is a weight factor and *t* the *t*-th propagation and ensembling iteration. As the number of iterations approaches infinity, we obtain $\lim_{t\to \infty }{\left(\omega \hat{A}\right)}^{t}=0$ and $\lim_{t\to \infty }\sum_{i=0}^{t-1}{\left(\omega \hat{A}\right)}^{i}={\left(I-\omega \hat{A}\right)}^{-1}$; hence an approximate inference formulation can be obtained as follows:

(9) $$Q^{T}=\left(1-\omega \right){\left(I-\omega \hat{A}\right)}^{-1}P^{T}.$$

Thereafter, the optimized feature representation, $Q^{T}$, and $P^{S}$ are transformed into Equations (2) and (3) to calculate $L_{\mathrm{FD}}$ = $L_{\mathrm{WKD}}$. By computing the feature-distillation loss $L_{\mathrm{FD}}$, we facilitate the acquisition of robust feature representations while minimizing the deviation from those learned in the first stage.

Next, our introduction of the LR-based approaches and WKD-BKE-integrated feature distillation into the CSSL framework enables the encoder to effectively capture the relationships between newly acquired data and previously learned knowledge. This integration mitigates the effects of catastrophic forgetting during pretraining as well as facilitating the learning of richer, more robust feature representations. Following the three-stage CSSL procedure, the ViT encoder $\phi_{M_{1}}$ is fine-tuned on a separate labeled dataset for downstream tasks, such as classification. During fine-tuning, $\phi_{M_{1}}$ is integrated with a randomly initialized task-specific multi-layer perceptron head and applied to the downstream tasks. Algorithm 1 summarizes the proposed CSSL framework.
**Algorithm 1** Algorithm of the proposed CSSL framework.**Input:** $\left\{D_{1},D_{2}\right\}$: two subsets from different domains, *B*: memory buffer, $T_{M_{1}},T_{M_{2}}$: tokenizers,      $\phi_{M_{1}},\phi_{M_{2}}$: encoders, $\psi_{M_{1}},\psi_{M_{2}}$: model-specific decoders, *K*-means(·): *k*-means clustering      operation, ClusterSample(·): operation for sampling cluster centers, LatentReplay(·): LR      operation **Output:** $\phi_{M_{2}}$, $T_{M_{2}}$
      **Stage 1: SSL on $D_{1}$**
1:   Set the training dataset: $D\leftarrow D_{1}$
2:   Update $\phi_{M_{1}}$, $T_{M_{1}}$, and $\psi_{M_{1}}$ by minimizing $L_{\mathrm{SSL}}$, following Equation (1)
      **Stage 2: Sampling Features into the Memory Buffer**
3:   Obtain clusters: $C\leftarrow K-Means(\phi_{M_{1}}\left(D_{1}\right))$
4:   Populate the memory buffer: B←ClusterSample(C)
      **Stage 3: CSSL with Feature Distillation on $D_{2}$**
5:   Set the training dataset: $D\leftarrow D_{2}$
6:   Extract the mini-batch feature representations: $P^{S}\leftarrow \phi_{M_{2}}\left(D_{2}\right)$
7:   Retrieve replayed feature representations from *B*: $P^{T}\leftarrow LatentReplay\left(B\right)$
8:   Obtain $Q^{T}$ by calculating the similarity between $P^{S}$ and $P^{T}$, following Equations (5)–(9)
9:   Update $\phi_{M_{2}}$, $T_{M_{2}}$, and $\psi_{M_{2}}$ by minimizing $L_{\mathrm{SSL}}$ and $L_{\mathrm{FD}}$ with $Q^{T}$ and $P^{S}$, following      Equations (1) and (4), respectively.


## 4. Experiments

We comprehensively experiment on classification tasks to validate the effectiveness of the proposed CSSL framework. These experiments include ablation studies, hyperparameter analyses, and an investigation of the impact of extending pretraining stages. The dataset and experimental settings are introduced in Section 4.1. Additionally, the classification-task performances with different pretraining datasets are discussed in Section 4.2. Furthermore, the impacts of hyperparameters on the experimental results are presented in Section 4.3. The ablation study of the proposed CSSL framework is discussed in Section 4.4. Finally, the impact of extending the pretraining stages in CSSL is demonstrated in Section 4.5.

### 4.1. Datasets and Settings

**Figure 2:**
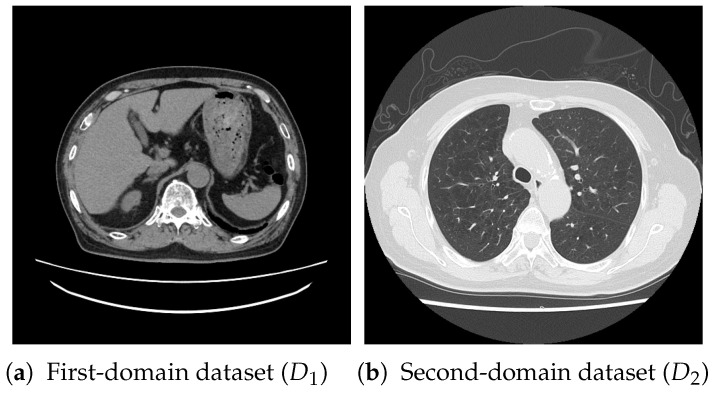
Examples of chest CT images on the subsets from the J-MID database: (**a**) first-domain dataset ($D_{1}$) and (**b**) second-domain dataset ($D_{2}$).

**Figure 3:**
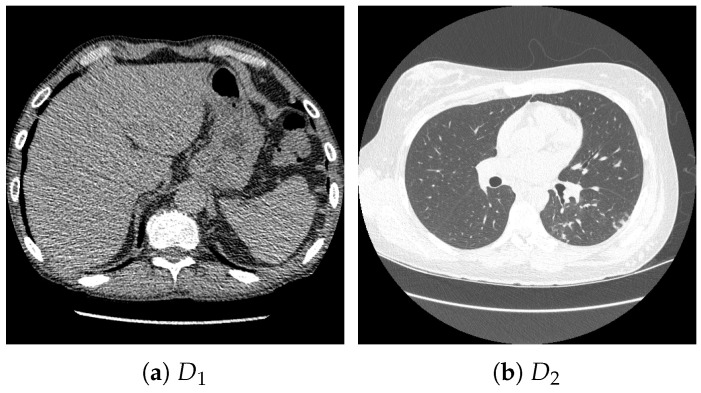
Examples of chest CT images on the subset from the RICORD dataset: (**a**) $D_{1}$ and (**b**) $D_{2}$.

**Figure 4:**
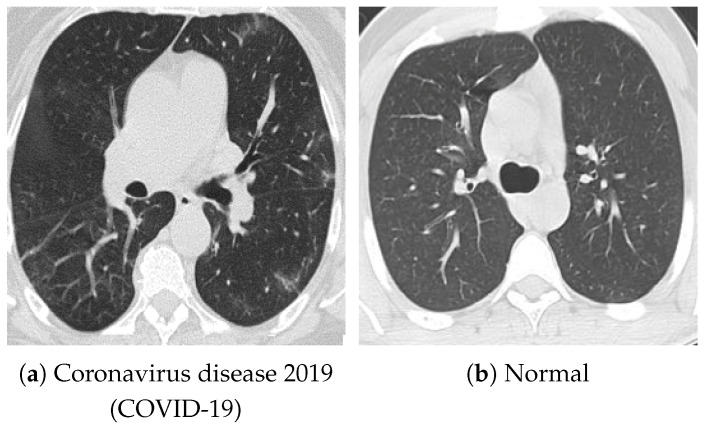
Examples of chest CT images on the SARS-CoV-2 CT-Scan dataset: (**a**) COVID-19 and (**b**) Normal.

**Figure 5:**
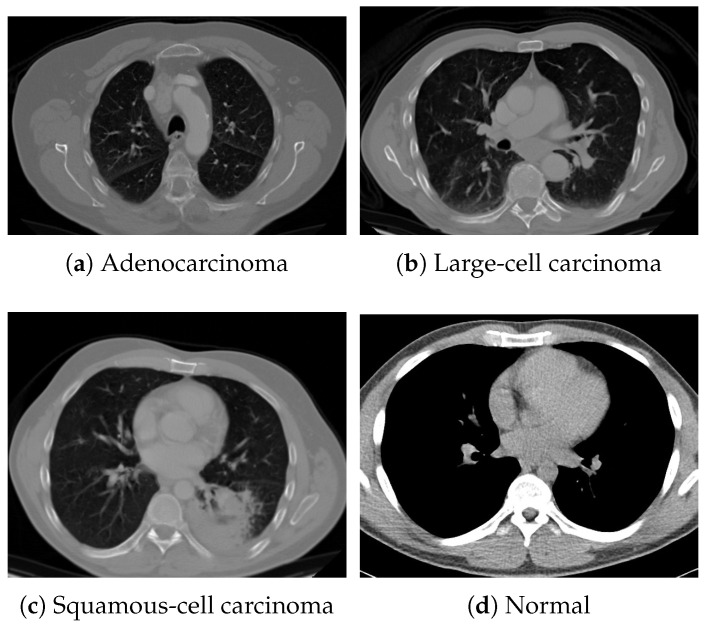
Examples of chest CT images from the Chest CT-Scan Images dataset: (**a**) adenocarcinoma, (**b**) large-cell carcinoma, (**c**) squamous-cell carcinoma, and (**d**) normal.

For pretraining, we utilized a subset of the J-MID (https://www.radiology.jp/j-mid/ (accessed on 6 April 2025)) database, which contains large-scale CT scans from Japanese medical institutions, and the RICORD dataset [58], an open dataset that was developed collaboratively by the Radiological Society of North America and international partners and contains chest CT scans collected from four countries. Each dataset was constructed with two domains based on mediastinal and lung window settings in chest CT images. Both domains are denoted as $D_{1}$ and $D_{2}$, and the labels are not used during pretraining. Specifically, for the J-MID subset, $D_{1}$ (the mediastinal window) contains 31,256 CT images, and $D_{2}$ (the lung window) contains 26,403 CT images. The RICORD dataset comprises 12,897 $D_{1}$ (mediastinal window) images and 11,668 $D_{2}$ (lung window) images for pretraining. For the J-MID dataset, $D_{1}$ was generated using a window level (WL) of 40±20 HU and a window width (WW) of 350±50 HU, whereas $D_{2}$ was generated using a WL of −625±75 HU and a WW of 1350±350 HU. For the RICORD dataset, $D_{1}$ was generated with a WL of 40±20 HU and a WW of 350±50 HU, while $D_{2}$ was generated with a WL of −600±100 HU and a WW of 1300±300 HU. These parameter ranges were selected to cover clinically standard lung and mediastinal window settings while accommodating inter-scan variability in DICOM metadata. The corresponding images for each example are shown in Figure 2 and Figure 3. For fine-tuning and evaluation, we utilized two public datasets: the SARS-CoV-2 CT-Scan Dataset [59] and the Chest CT-Scan Images Dataset (https://www.kaggle.com/datasets/mohamedhanyyy/chest-ctscan-images (accessed on 6 April 2025)). Both datasets were used for the coronavirus disease 2019 (COVID-19) and chest cancer classification tasks, respectively. The data breakdown is as follows: the SARS-CoV-2 CT-Scan Dataset comprises 1589 training, 397 validation, and 495 test images, labeled into two (COVID-19 and Normal) classes. The Chest CT-Scan Images Dataset comprises 490 training, 123 validation, and 315 test images labeled into four (adenocarcinoma, large-cell carcinoma, squamous-cell carcinoma, and normal) classes. COVID-19 classification and lung cancer classification were selected as downstream tasks because chest CT is widely used in clinical practice for diagnosing both COVID-19 and lung cancer, allowing for an evaluation that closely reflects real-world clinical scenarios. Accordingly, to ensure reproducibility and enable fair comparisons with prior studies, we prioritized the use of publicly available datasets. The corresponding images for each example are shown in Figure 4 and Figure 5.

In the pretraining of the MAE, the batch sizes were set to 64 and 32 in the first and third stages, respectively, and the masking ratio, *r*, was set to 0.75, with ViT-B [52] being deployed as the encoder. Next, augmentation techniques, such as random crop, resize, and flip, were applied to the images. Additionally, a warm-up strategy was applied during the first 40 epochs, gradually increasing the learning rate from 0 to 0.00015. Subsequently, the learning rate was reduced to 0 via a cosine schedule. For *k*-means sampling, the parameters, α and β, which determine the sampling ratio, were set to 0.01 and 0.05, respectively [26]. Notably, γ, which adjusts the contributions of the mean and covariance in the WKD loss, $L_{\mathrm{WKD}}$, was set to 2.0 and 3.0 during the pretraining on the J-MID subset and RICORD dataset, respectively. In BKE, the hyperparameter, ω, was set to 0.5 [60,61,62]. The AdamW optimizer [63] was utilized, with the learning rate set to 0.00005. Pretraining and fine-tuning were conducted for 300 and 80 epochs per stage, respectively.

For the evaluation metrics, we employed three metrics: two-class classification accuracy (ACC), the area under the receiver operating characteristic curve (AUC), and the F1-score (F1). To ensure robustness, we averaged the results across three of the four random seeds (0, 10, 100, and 1000). In all tables, the best performance is highlighted in bold for each experimental result. To evaluate the effectiveness of our method, we compared it with the following approaches: the state-of-the-art CSSL method for medical imaging, MedCoSS [26], MAE [53] simultaneously pretrained on $D_{1}$ and $D_{2}$, MAE pretrained only on $D_{1}$, and MAE pretrained only on $D_{2}$. As a baseline method, we employed a model that was fine-tuned without MAE-based self-supervised pretraining.

### 4.2. Classification-Task Performance with Different Pretraining Datasets

Table 1 presents the classification results obtained after pretraining on the J-MID subset and evaluating on the SARS-CoV-2 CT-Scan dataset (for COVID-19 classification) and the Chest CT-Scan Images dataset (for lung cancer classification). Furthermore, to examine the effectiveness of the domain-pretraining order, we performed continual pretraining by interchanging domains $D_{1}$ and $D_{2}$. Notably, the highest accuracy on the SARS-CoV-2 CT-Scan dataset was achieved when continual pretraining was performed from $D_{1}$ to $D_{2}$, whereas the best performance on the Chest CT-Scan Images dataset was obtained when the pretraining order was reversed from $D_{2}$ to $D_{1}$. These results suggest that the domain-pretraining order in continual pretraining is associated with variations in downstream task performance.

Such order dependence is considered to be related to the effects of catastrophic forgetting, which is a well-known phenomenon in SCL and CSSL. Prior studies have reported that the feature space learned by deep models is influenced by parameter updates in later training stages, resulting in learned representations that tend to be biased toward data encountered more recently [64,65]. In particular, when SCL is performed without strict constraints, parameter updates in later stages can reorganize the embedding space. This reorganization can lead to the partial overwriting of representations acquired in earlier stages. Consequently, the final learned representations tend to align with the statistical characteristics of the domain learned last. In our experimental setting, similar tendencies were observed. When a domain that is semantically closer to the downstream task was presented at the final stage, the learned representations exhibited relatively higher transfer performance. In contrast, when the final domain was weakly related to the downstream task, the learned representations tended to deviate from features that are useful for the downstream task. As a result, performance degradation was observed.

When pretrained on the same-order domains, the proposed method consistently outperformed MedCoSS across all evaluation metrics. Although the LR mechanism and the WKD-BKE-integrated feature distillation introduced in this study do not eliminate the effects of catastrophic forgetting, the results indicate that they mitigate its negative impact. Furthermore, under the joint-learning scenario in which both domains were pretrained simultaneously, the proposed method also surpassed the MAE baseline. These findings suggest that, in CSSL, appropriately designed continual pretraining is more effective than simultaneous pretraining in reducing domain interference. In addition, Table 2 presents the results obtained after pretraining on the RICORD dataset. Except for the continual pretraining order from $D_{1}$ to $D_{2}$, these results are largely consistent with the trends observed on the J-MID dataset. Overall, these results demonstrate that the proposed LR-based CSSL framework effectively alleviates data interference during continual pretraining.

In addition to performance evaluation, we analyzed the computational cost of the proposed method. During Stage 1 pretraining, the GPU memory consumption is 7731 MB, and the training time per epoch is 49.78 s. During Stage 3 continual pretraining, the GPU memory consumption is 5956 MB, and the training time per epoch is 59.94 s. Moreover, the inference time per epoch during the test phase of fine-tuning is 3.56 s. In our experiments, we used ViT-B as the backbone, resulting in a model with 86 M parameters. The batch size was set to 64 during Stage 1 pretraining and 32 during Stage 3 continual pretraining. With respect to processing requirements in hospital settings, the proposed framework can be deployed on standard GPU-equipped medical servers. In our experiments, conducted using an NVIDIA GeForce RTX 4090 with approximately 24 GB of GPU memory, the proposed method maintained a moderate model size and GPU memory footprint. These results indicate that the proposed method enables efficient incremental updates without imposing excessive computational or memory overhead, making it suitable for deployment in real-world clinical environments.

### 4.3. Impact of Hyperparameters on the Experimental Results

To investigate feature distillation for mitigating data interference and handling distributional differences across stages, we explored optimal parameter settings to minimize deviations from the knowledge acquired in the previous stage during subsequent learning. Specifically, we examined the hyperparameter γ in the WKD loss, $L_{\mathrm{WKD}}$, and the batch size of BKE to determine their optimal values. The evaluation was on the COVID-19 classification task using the SARS-CoV-2 CT-Scan dataset.

In the proposed method, the hyperparameter in the WKD loss represents γ, which controls the relative contributions of the mean and covariance terms. Table 3 presents the classification results obtained with varying γ values when pretraining was performed on the J-MID subset. Table 4 presents the classification results with varying γ values when pretraining was performed on the RICORD dataset. For the proposed method, the optimal γ setting was 2.0 for the $D_{2}$–$D_{1}$ continual pretraining order and 3.0 for the $D_{1}$ to $D_{2}$ order. Accordingly, as γ increases, the mean term in the WKD loss exerts more significant influence, indicating that the mean plays a more crucial role than the covariance.

Next, in the BKE of the proposed method, knowledge is propagated and ensembled based on the affinity between the feature representations replayed from the *B* and those generated by $M_{2}$ within mini-batches in the third learning stage. Table 5 and Table 6 present the classification results with varying batch sizes when pretraining was performed on the J-MID subset and RICORD dataset, respectively. Notably, the optimal results were obtained with a batch size of 32 regardless of the utilized dataset. This behavior is closely related to the characteristics of the BKE. When the batch size is small, the diversity of feature representations jointly considered within BKE becomes limited. As a result, the estimation of batch-level feature statistics becomes unstable, potentially reducing the effectiveness of knowledge transfer. In contrast, when the batch size is large, the regularization induced by previously learned feature distributions becomes relatively stronger, which may bias the learned representations toward existing knowledge.

Overall, these findings indicate that the proposed method maintains robustness across a reasonable range of hyperparameter settings. The observed performance-variation trends with respect to the mean–covariance balance and batch size are consistent and interpretable, indicating that the proposed framework behaves stably and predictably under different configurations. This robustness demonstrates its practicality and reliability for continual pretraining across diverse medical-imaging domains.

### 4.4. Ablation Studies

To evaluate the effectiveness of the proposed LR-based and WKD-BKE-integrated feature-distillation approaches, we performed an ablation study on the SARS-CoV-2 CT-Scan dataset, and Table 7 and Table 8 present the results when pretraining was performed on the J-MID subset and RICORD dataset, respectively. The first row reveals the performance of the baseline, which adopts an experience-replay-based approach with a *k*-means sampling strategy as well as performs feature distillation using only the mean-squared-error loss. The last row highlights the performance of the proposed method. We confirmed that replacing the experience-replay-based approach with LR in the proposed CSSL framework improved classification accuracy, as LR eliminated the dependence on raw image storage by replaying latent representations, thereby reducing the noise and redundancy that are inherent in pixel-level data. Consequently, the model retained informative and domain-invariant features more effectively, enhancing stability and knowledge retention during continual pretraining. Furthermore, although integrating LR with only WKD or BKE did not yield significant improvement, its incorporation with both techniques yielded substantial performance improvements. Specifically, WKD aligns the feature distributions between past and newly acquired representations to suppress domain-specific biases. Conversely, BKE exploits the similarity among feature representations within mini-batches and those replayed from the memory buffer *B*, facilitating feature-level knowledge propagation as well as stabilizing the optimization process. Additionally, their integration offers complementary benefits, where WKD preserves consistency across domains, and BKE promotes coherence within batches. This synergy enables the model to achieve more robust feature representations and enhanced classification accuracy.

Thus, the newly introduced feature distillation enhances the knowledge-retention capability of the model while maintaining its new-information adaptability. Overall, these results demonstrate the effectiveness of each component of the proposed method, underscoring their roles in mitigating data interference and improving continual-pretraining performance within the CSSL framework.

### 4.5. Impact of Stage Extension on Continual Pretraining

To examine the effect of progressive domain expansion during pretraining, we conducted four-stage continual pretraining on the RICORD dataset and J-MID subset, evaluating the resulting models on the SARS-CoV-2 CT-Scan dataset. The selected sequential training model was as follows: $D_{1}$–$D_{2}$ (RICORD), followed by $D_{1}$–$D_{2}$ (J-MID). This sequence was selected based on the results in Table 1 and Table 2, which indicate that models pretrained on the RICORD dataset achieved lower accuracy on the SARS-CoV-2 CT-Scan task compared with those pretrained on the J-MID subset. Therefore, we attempted to improve generalizability by first pretraining on the domains from the RICORD dataset before progressively expanding the pretraining to domains from the J-MID subset.

Table 9 summarizes the results of this extended-pretraining experiment. In Stage 1, where the model was pretrained only on RICORD $D_{1}$, it exhibited limited performance on the SARS-CoV-2 CT-Scan classification task. In Stage 2, further pretraining on RICORD $D_{2}$ significantly improved all metrics, particularly the AUC, indicating enhanced representation robustness through pretraining on multiple domains within RICORD. In Stage 3, where the model was further trained on J-MID $D_{1}$, its performance decreased slightly owing to the domain characteristics, as this $D_{1}$ is less similar to the target SARS-CoV-2 CT-Scan dataset, whereas $D_{2}$ shares more common features with the target domain. In Stage 4, following the incorporation of J-MID $D_{2}$, the model recovered and exhibited improved performance.

Overall, these findings indicate that extending the pretraining stages enables the model to learn more diverse and transferable representations, which consequently improve its performance on the downstream task. These findings reveal that our approach can continually learn from images acquired from different domains across multiple medical institutions while preserving data privacy, thereby enabling more accurate, high-performance diagnostic capabilities.

## 5. Discussion

Our experimental results demonstrated the effectiveness and robustness of our method across two distinct domains of unlabeled chest CT images, namely the mediastinal and lung windows, using two publicly available datasets (J-MID and RICORD). The proposed framework outperformed existing SSL and CSSL approaches, exhibiting notable advantages. A major strength of the proposed method is its privacy-preserving design: the framework adopts LR, storing intermediate feature representations instead of original CT images. This strategy enables continual pretraining while eliminating the risk of sensitive data leakage, making the design suitable for dynamic clinical environments where imaging conditions and acquisition protocols change continually. The integration of LR with WKD-BKE was crucial to achieving an adaptation–retention balance. Our ablation studies confirmed that LR mitigated catastrophic forgetting by simultaneously retaining essential latent features and maintaining privacy protection. Moreover, the synergy between WKD-BKE exerted complementary effects. WKD aligned feature distributions across different domains, reducing domain-specific bias, whereas BKE stabilized the training process by promoting feature-level knowledge propagation among mini-batches. Collectively, these mechanisms facilitated the learning of representations that were both domain invariant and discriminative, thereby improving performance in the downstream classification task.

Despite these results, several challenges exist. A critical issue in continual pretraining is the significant dependence of model performance on the domain-exposure order: performance improves when the final domain is closely related to the evaluation data, decreasing otherwise. Therefore, for clinical applications, the relationship between the pretraining data used and the target images must be clarified before applying the model. One direction for future work is the visualization of latent feature distributions under different domain-exposure orders. Such qualitative analyses may provide deeper insights into how the learned feature space evolves during CSSL. They may also clarify how later training stages influence the structure of the embedding space, leading to a more interpretable understanding of the observed order-dependent behavior. We also note that the current evaluation focuses on downstream tasks that are relatively close to the pretraining domains. As a next step, we plan to evaluate the proposed method on datasets exhibiting diverse domain shifts, such as inter-institutional variations across scanner vendors, reconstruction kernels, and noise levels. This evaluation will help clarify the generalizability of the proposed CSSL framework under more realistic and challenging conditions. Additionally, although the proposed method stores only representative latent features in *B*, memory management remains challenging in long-term continual pretraining as the number of domains increases. Future work may explore adaptive feature compression and dynamic memory-management strategies to improve efficiency. Closely related to this issue is the design choice of feature representations used for LR. In this study, LR uses features from the final encoder layer of a pretrained ViT, which capture high-level semantic information, while representations from earlier or intermediate layers tend to preserve local structural cues. The use of intermediate or multi-layer feature representations is left for future work. Furthermore, because the proposed framework explicitly stores and reuses latent feature representations, security considerations become important, especially in medical imaging. Although intermediate features are stored to retain knowledge from past domains, such representations may be vulnerable to feature inversion attacks [66]. As future work, we consider representing past-domain knowledge as statistical distributions in the latent feature space and performing LR using sampled features [67,68]. This design avoids storing raw images or sample-level feature vectors and is expected to improve robustness against feature inversion attacks.

Beyond these considerations, federated learning (FL) enables model training in distributed environments without centralizing data and has gained attention for privacy-preserving medical image analysis [69,70]. However, handling temporal changes in data distributions remains a challenge in federated settings. Recent studies have shown that integrating FL with SCL is a promising direction to address non-stationarity arising from evolving client data distributions [71,72]. Extending these insights to CSSL, we consider the integration of FL with CSSL as future work to support privacy-preserving adaptation to dynamic and heterogeneous data environments. We plan to address these limitations by further advancing the CSSL framework to improve scalability, robustness, and adaptability. Such extensions are expected to enhance generalization across diverse and evolving data distributions, ultimately enabling the development of more reliable and practical medical imaging models for real-world clinical deployment.

## 6. Conclusions

We proposed a privacy-aware CSSL framework to address the domain shifts in medical imaging. The method incorporates an LR mechanism with WKD-BKE-integrated feature distillation, thus effectively mitigating catastrophic forgetting and preserving data privacy simultaneously. Our experiments on multi-window chest-CT datasets demonstrated that our approach outperformed existing state-of-the-art SSL and CSSL methods, achieving superior robustness and generalizability across domains. However, several limitations persisted, including the dependence on domain-exposure order, challenges in long-term memory management, and considerations related to feature representation design and security. We plan to address these limitations by further extending the CSSL framework toward more scalable and robust learning to establish more generalizable and privacy-preserving medical artificial intelligence systems.

## Figures and Tables

**Table 1:** Experimental results of the proposed method and conventional state-of-the-art methods pretrained on the J-MID subset.

		SARS-CoV-2 CT-Scan Dataset			Chest CT-Scan Images Dataset		
**Method**	**Domain**	**ACC**	**AUC**	**F1**	**ACC**	**AUC**	**F1**
Ours	$D_{1}$ → $D_{2}$	$0.873_{\pm 0.030}$	$0.953_{\pm 0.012}$	$0.873_{\pm 0.030}$	$0.716_{\pm 0.042}$	$0.943_{\pm 0.002}$	$0.698_{\pm 0.054}$
MedCoSS [26]	$D_{1}$ → $D_{2}$	$0.858_{\pm 0.020}$	$0.940_{\pm 0.016}$	$0.858_{\pm 0.020}$	$0.662_{\pm 0.020}$	$0.911_{\pm 0.008}$	$0.642_{\pm 0.015}$
Ours	$D_{2}$ → $D_{1}$	$0.777_{\pm 0.010}$	$0.857_{\pm 0.016}$	$0.777_{\pm 0.010}$	$0.833_{\pm 0.007}$	$0.965_{\pm 0.004}$	$0.845_{\pm 0.007}$
MedCoSS [26]	$D_{2}$ → $D_{1}$	$0.763_{\pm 0.022}$	$0.847_{\pm 0.024}$	$0.761_{\pm 0.024}$	$0.822_{\pm 0.033}$	$0.962_{\pm 0.009}$	$0.833_{\pm 0.034}$
MAE [53]	$D_{1}$ + $D_{2}$	$0.784_{\pm 0.027}$	$0.871_{\pm 0.025}$	$0.783_{\pm 0.027}$	$0.807_{\pm 0.043}$	$0.964_{\pm 0.008}$	$0.804_{\pm 0.057}$
MAE [53]	$D_{1}$	$0.729_{\pm 0.015}$	$0.804_{\pm 0.014}$	$0.724_{\pm 0.015}$	$0.665_{\pm 0.061}$	$0.920_{\pm 0.003}$	$0.668_{\pm 0.063}$
MAE [53]	$D_{2}$	$0.760_{\pm 0.024}$	$0.831_{\pm 0.041}$	$0.756_{\pm 0.026}$	$0.519_{\pm 0.037}$	$0.874_{\pm 0.011}$	$0.509_{\pm 0.036}$
Baseline	None	$0.620_{\pm 0.041}$	$0.644_{\pm 0.050}$	$0.599_{\pm 0.055}$	$0.495_{\pm 0.067}$	$0.801_{\pm 0.023}$	$0.500_{\pm 0.060}$

**Table 2:** Experimental results of the proposed method and conventional state-of-the-art methods pretrained on the RICORD dataset.

		SARS-CoV-2 CT-Scan Dataset			Chest CT-Scan Images Dataset		
**Method**	**Domain**	**ACC**	**AUC**	**F1**	**ACC**	**AUC**	**F1**
Ours	$D_{1}$ → $D_{2}$	$0.844_{\pm 0.034}$	$0.936_{\pm 0.023}$	$0.843_{\pm 0.034}$	$0.689_{\pm 0.103}$	$0.927_{\pm 0.031}$	$0.672_{\pm 0.148}$
MedCoSS [26]	$D_{1}$ → $D_{2}$	$0.815_{\pm 0.045}$	$0.911_{\pm 0.027}$	$0.814_{\pm 0.046}$	$0.741_{\pm 0.061}$	$0.952_{\pm 0.019}$	$0.725_{\pm 0.079}$
Ours	$D_{2}$ → $D_{1}$	$0.749_{\pm 0.020}$	$0.821_{\pm 0.024}$	$0.745_{\pm 0.021}$	$0.868_{\pm 0.014}$	$0.980_{\pm 0.002}$	$0.883_{\pm 0.014}$
MedCoSS [26]	$D_{2}$ → $D_{1}$	$0.748_{\pm 0.027}$	$0.814_{\pm 0.025}$	$0.744_{\pm 0.029}$	$0.818_{\pm 0.059}$	$0.973_{\pm 0.007}$	$0.827_{\pm 0.062}$
MAE [53]	$D_{1}$ + $D_{2}$	$0.760_{\pm 0.026}$	$0.844_{\pm 0.027}$	$0.757_{\pm 0.030}$	$0.709_{\pm 0.047}$	$0.940_{\pm 0.007}$	$0.717_{\pm 0.054}$
MAE [53]	$D_{1}$	$0.727_{\pm 0.023}$	$0.795_{\pm 0.007}$	$0.722_{\pm 0.023}$	$0.614_{\pm 0.028}$	$0.899_{\pm 0.016}$	$0.632_{\pm 0.030}$
MAE [53]	$D_{2}$	$0.768_{\pm 0.008}$	$0.843_{\pm 0.026}$	$0.765_{\pm 0.010}$	$0.474_{\pm 0.056}$	$0.854_{\pm 0.010}$	$0.465_{\pm 0.063}$
Baseline	None	$0.587_{\pm 0.019}$	$0.611_{\pm 0.017}$	$0.557_{\pm 0.030}$	$0.516_{\pm 0.037}$	$0.799_{\pm 0.026}$	$0.499_{\pm 0.061}$

**Table 3:** Evaluation results on the SARS-CoV-2 CT-Scan dataset using the J-MID subset with varying hyperparameters, γ. Batch size was fixed at 32.

Domain	γ	ACC	AUC	F1
$D_{1}$ → $D_{2}$	0.0	$0.844_{\pm 0.015}$	$0.932_{\pm 0.013}$	$0.844_{\pm 0.015}$
	1.0	$0.852_{\pm 0.012}$	$0.937_{\pm 0.017}$	$0.852_{\pm 0.012}$
	2.0	$0.873_{\pm 0.030}$	$0.953_{\pm 0.012}$	$0.873_{\pm 0.030}$
	3.0	$0.852_{\pm 0.009}$	$0.939_{\pm 0.011}$	$0.852_{\pm 0.009}$
	4.0	$0.840_{\pm 0.023}$	$0.935_{\pm 0.019}$	$0.840_{\pm 0.023}$
$D_{2}$ → $D_{1}$	0.0	$0.747_{\pm 0.016}$	$0.843_{\pm 0.012}$	$0.745_{\pm 0.016}$
	1.0	$0.752_{\pm 0.015}$	$0.844_{\pm 0.027}$	$0.751_{\pm 0.015}$
	2.0	$0.777_{\pm 0.010}$	$0.857_{\pm 0.016}$	$0.777_{\pm 0.010}$
	3.0	$0.774_{\pm 0.010}$	$0.863_{\pm 0.008}$	$0.774_{\pm 0.010}$
	4.0	$0.762_{\pm 0.021}$	$0.849_{\pm 0.021}$	$0.761_{\pm 0.015}$

**Table 4:** Evaluation results on the SARS-CoV-2 CT-Scan dataset for the model pretrained using the RICORD dataset with varying γ. Batch size was fixed at 32.

Domain	γ	ACC	AUC	F1
$D_{1}$ → $D_{2}$	0.0	$0.791_{\pm 0.041}$	$0.913_{\pm 0.030}$	$0.787_{\pm 0.042}$
	1.0	$0.804_{\pm 0.005}$	$0.904_{\pm 0.014}$	$0.803_{\pm 0.007}$
	2.0	$0.801_{\pm 0.050}$	$0.901_{\pm 0.017}$	$0.798_{\pm 0.055}$
	3.0	$0.844_{\pm 0.034}$	$0.936_{\pm 0.023}$	$0.843_{\pm 0.034}$
	4.0	$0.832_{\pm 0.015}$	$0.916_{\pm 0.011}$	$0.831_{\pm 0.016}$
$D_{2}$ → $D_{1}$	0.0	$0.749_{\pm 0.008}$	$0.822_{\pm 0.005}$	$0.746_{\pm 0.009}$
	1.0	$0.743_{\pm 0.018}$	$0.823_{\pm 0.018}$	$0.742_{\pm 0.033}$
	2.0	$0.739_{\pm 0.012}$	$0.820_{\pm 0.015}$	$0.735_{\pm 0.015}$
	3.0	$0.749_{\pm 0.020}$	$0.821_{\pm 0.024}$	$0.745_{\pm 0.021}$
	4.0	$0.757_{\pm 0.019}$	$0.833_{\pm 0.034}$	$0.755_{\pm 0.020}$

**Table 5:** Evaluation results on the SARS-CoV-2 CT-Scan dataset for the model pretrained using the J-MID subset with varying batch sizes. γ was fixed at 2.0.

Domain	Batch Size	ACC	AUC	F1
$D_{1}$ → $D_{2}$	16	$0.839_{\pm 0.019}$	$0.935_{\pm 0.008}$	$0.839_{\pm 0.019}$
	32	$0.873_{\pm 0.030}$	$0.953_{\pm 0.012}$	$0.873_{\pm 0.030}$
	64	$0.858_{\pm 0.027}$	$0.933_{\pm 0.018}$	$0.858_{\pm 0.027}$
	128	$0.865_{\pm 0.009}$	$0.944_{\pm 0.008}$	$0.865_{\pm 0.009}$
$D_{2}$ → $D_{1}$	16	$0.748_{\pm 0.009}$	$0.841_{\pm 0.006}$	$0.745_{\pm 0.030}$
	32	$0.777_{\pm 0.016}$	$0.857_{\pm 0.010}$	$0.777_{\pm 0.010}$
	64	$0.743_{\pm 0.017}$	$0.829_{\pm 0.006}$	$0.741_{\pm 0.006}$
	128	$0.739_{\pm 0.010}$	$0.825_{\pm 0.013}$	$0.737_{\pm 0.013}$

**Table 6:** Evaluation results on the SARS-CoV-2 CT-Scan dataset for the model pretrained on the RICORD dataset with varying batch sizes. γ was fixed at 3.0.

Domain	Batch Size	ACC	AUC	F1
$D_{1}$ → $D_{2}$	16	$0.793_{\pm 0.037}$	$0.868_{\pm 0.031}$	$0.792_{\pm 0.038}$
	32	$0.844_{\pm 0.034}$	$0.936_{\pm 0.023}$	$0.843_{\pm 0.034}$
	64	$0.832_{\pm 0.018}$	$0.930_{\pm 0.010}$	$0.832_{\pm 0.018}$
	128	$0.831_{\pm 0.031}$	$0.918_{\pm 0.020}$	$0.830_{\pm 0.031}$
$D_{2}$ → $D_{1}$	16	$0.729_{\pm 0.005}$	$0.802_{\pm 0.003}$	$0.725_{\pm 0.003}$
	32	$0.749_{\pm 0.024}$	$0.821_{\pm 0.020}$	$0.745_{\pm 0.021}$
	64	$0.743_{\pm 0.013}$	$0.813_{\pm 0.014}$	$0.741_{\pm 0.013}$
	128	$0.750_{\pm 0.009}$	$0.837_{\pm 0.015}$	$0.748_{\pm 0.010}$

**Table 7:** Results of the ablation studies on the latent replay (LR), Wasserstein distance (WD)-based knowledge distillation (WKD), and batch-knowledge ensemble (BKE) when the model was pretrained on the J-MID subset.

LR	WKD	BKE	ACC	AUC	F1
			$0.827_{\pm 0.020}$	$0.935_{\pm 0.001}$	$0.826_{\pm 0.021}$
✔			$0.833_{\pm 0.034}$	$0.936_{\pm 0.016}$	$0.832_{\pm 0.034}$
✔		✔	$0.827_{\pm 0.024}$	$0.929_{\pm 0.022}$	$0.826_{\pm 0.024}$
✔	✔		$0.845_{\pm 0.021}$	$0.934_{\pm 0.018}$	$0.845_{\pm 0.021}$
✔	✔	✔	$0.873_{\pm 0.030}$	$0.953_{\pm 0.012}$	$0.873_{\pm 0.030}$

**Table 8:** Results of the ablation studies of LR, WKD, and BKE when the model was pretrained on the RICORD dataset.

LR	WKD	BKE	ACC	AUC	F1
			$0.804_{\pm 0.047}$	$0.905_{\pm 0.019}$	$0.803_{\pm 0.048}$
✔			$0.826_{\pm 0.052}$	$0.906_{\pm 0.031}$	$0.824_{\pm 0.054}$
✔		✔	$0.840_{\pm 0.035}$	$0.927_{\pm 0.023}$	$0.839_{\pm 0.037}$
✔	✔		$0.822_{\pm 0.047}$	$0.906_{\pm 0.038}$	$0.820_{\pm 0.050}$
✔	✔	✔	$0.844_{\pm 0.034}$	$0.936_{\pm 0.023}$	$0.843_{\pm 0.034}$

**Table 9:** Experimental results for the extended pretraining stage. Four-stage continual pretraining was conducted sequentially on RICORD and J-MID domains $D_{1}$ and $D_{2}$.

Stage	ACC	AUC	F1
Stage 1	$0.727_{\pm 0.023}$	$0.795_{\pm 0.007}$	$0.722_{\pm 0.023}$
Stage 2	$0.844_{\pm 0.034}$	$0.936_{\pm 0.023}$	$0.843_{\pm 0.034}$
Stage 3	$0.754_{\pm 0.015}$	$0.849_{\pm 0.021}$	$0.754_{\pm 0.015}$
Stage 4	$0.857_{\pm 0.020}$	$0.936_{\pm 0.020}$	$0.857_{\pm 0.019}$

## Data Availability

The RICORD dataset, SARS-CoV-2 CT-Scan Dataset, and Chest CT-Scan Images Dataset used in this study are publicly available as cited in the notes and references. The J-MID database cannot be released.

## Abbreviations

The following abbreviations are used in this manuscript:

SL	Supervised Learning
SSL	Self-Supervised Learning
CT	Computed Tomography
CSSL	Continual Self-Supervised Learning
SCL	Supervised Continual Learning
LR	Latent Replay
NNs	Neural Networks
WD	Wasserstein Distance
WKD	Wasserstein distance-based Knowledge Distillation
BKE	Batch-Knowledge Ensemble
MRI	Magnetic Resonance Imaging
ULM	Ultrasound Localization Microscopy
MAE	Masked AutoEncoder
COVID-19	Coronavirus Disease 2019
ACC	Accuracy
AUC	Area Under the receiver operating characteristic Curve
F1	F1-score
FL	Federated Learning
